# Geographical Variation in Nutrient Intake between Urban and Rural Areas of Jiangsu Province, China and Development of a Semi-Quantitative Food Frequency Questionnaire for Middle-Aged Inhabitants

**DOI:** 10.2188/jea.13.80

**Published:** 2007-11-30

**Authors:** Ying-Ming Wang, Bao-Qing Mo, Toshiro Takezaki, Nahomi Imaeda, Mieko Kimura, Xin-Ru Wang, Kazuo Tajima

**Affiliations:** 1 Department of Nutrition and Food Science, Nanjing Medical University.; 2 Division of Epidemiology and Prevention, Aichi Cancer Center Research Institute.; 3 Nagoya City School of Nutrition.; 4 Takeda Life Science Research Center.; 5 Nanjing Medical University.

**Keywords:** food, nutrient, intake, semi-quantitative food frequency questionnaire, China

## Abstract

The intake of food and nutrients differs between urban and rural areas in China. To develop a practical semi-quantitative food frequency questionnaire to cover both the urban and rural areas, we conducted diet surveys and compared food and nutrient intake between the two areas. We recruited 198 urban and 214 rural healthy inhabitants aged 35-55 years, and performed diet surveys, using a 3-day weighed dietary record approach. The intake of 29 nutrients was calculated according to actual consumption of foods, with Standard Food Composition Tables for China and Japan. Then, contribution analysis and multiple regression analysis were employed to select food items covering up to a 90% contribution and a 0.90 R^2^ of coefficient of determination, respectively. Consumption of energy and carbohydrates was greater in the rural area, but mean protein intake was higher in the urban case. Values for total fat were greater for rural than for urban males, with animal fat as the major contributor. We finally selected 117 and 76 food items for the urban and rural semi-quantitative food frequency questionnaires, respectively, covering 18 and 27 nutrients constituting up to 90% of the nutrient intake. Further validity and reproducibility tests are now needed to assess their appropriateness for usage.

Diet is one of the major environmental factors related to chronic diseases, such as cardiovascular ailments, diabetes, and cancer,^1^^-^^3^ and geographical variation in food and nutrient intake plays an important role in determining incidences of these diseases. Variation between individuals allows us to estimate the disease risk with dietary factors, but only when accurate information on food and nutrient intake is available.^4^

At the present, there are five main methods employed to assess food and nutrient intake: (1) food record; (2) 24-hour dietary recall; (3) food frequency; (4) brief dietary assessment, using a short questionnaire on food frequency or dietary habits; and (5) chemical analysis of food samples collected as duplicate meals.^4^^-^^6^ There are limitations with all but the food frequency approach in that the data obtained only represent diet intake for a short period of time, such as one to seven days, which may not be representative of longer-term consumption. Estimation of food frequency is therefore a useful tool to assess food intake over relatively long time periods, because it is designed to estimate the respondent’s usual intake of foods and to avoid bias due to transient variation in dietary intake. The cost of data collection and processing, and the respondent’s burden, are also relatively low. However, there is a disadvantage with this approach that detailed information may not be obtained and quantification is not as accurate as with recall and record methods. One reason for inaccuracies with the food frequency method is incomplete listing of major foods and serving sizes, in addition to recall bias. Therefore, development of a food list which sufficiently covers absolute amounts and between-person variation in food and nutrient intake is critical for obtaining accurate data by the food frequency method.

Chinese consume a variety of foods every day. The third Nationwide Survey for food and nutrient intake among inhabitants of China was conducted in 1992, and revealed geographical variation in intake between urban and rural areas.^7^ The evidence indicates that semi-quantitative food frequency questionnaires (SQFFQs) should cover urban and rural populations, in order to assess relationships between diet and disease risk, because recruited cases may comprise both populations. To develop SQFFQs for the assessment of cancer risk with dietary factors in the study regions, we conducted a survey for food and nutrient intake, using a 3-day dietary record method in Nanjing (urban) and Huai’an (rural) of Jiangsu Province, China, then making an objective comparison of the two cases.

## METHODS

### Study subjects

Huai’an is a rural city, located in the north of Jiangsu Province, with a population of approximately 1.2 million. We randomly recruited 214 healthy residents (102 males and 112 females) aged 35-55 years in April, 2000. Nanjing is the capital of Jiangsu Province, located in the middle, with 6 million inhabitants. We had conducted a dietary survey for randomly recruited healthy residents in Nanjing in 1995 and the present data were obtained from the subjects in this survey, comprising 94 males and 104 females aged 35-55 years who were randomly selected according to age and sex distribution. We determined the number of study subjects as 200 with 3-day dietary records in each area, because previous Japanese study comprised 351 subjects with one-day records for the development of SQFFQ.^8^ We also randomly recruited 50 inhabitants (25 males and 25 females) of Nanjing in 2000 to compare differences of nutrient intake by year.

At first, we selected households from four villages of the rural area and from four blocks of the urban area with middle economical condition, using residential records. Then, we selected one subject from family members, using random numbers. The sampling fractions were 178 per million for the rural area and 33 per million for urban area. Local staffs of public health center asked them to participate the survey after an oral explanation. All subjects agreed to participate in the survey, because they were informed that detailed guidance for healthy diet would be provided. Most of respondents, except 2 interviewees, could actually obtain this information after the survey.

### Dietary assessment

The dietary data for rural inhabitants were collected with 3-day weighed records (two weekdays and one weekend day) in 2000. The data for urban inhabitants were selected from 3-day weighed records (two weekdays and one weekend day) which were collected as 7-day weighed records in 1995. In both areas, investigators visited each subject, and weighed all materials before cooking three times per day for 3 or 7 days. Investigators checked all data recorded within 24 hours of the survey, and some of them were again re-checked by a supervisor.

### Nutrient intake analyses

Nutrient intake was calculated by multiplying the food intake by nutrient content, using the Standard Food Composition Table (first edition) authored by the Nutrition and Food Hygiene Institute of the Preventive Science Academy in China.^9^ We also used the data in the Japanese Standard Tables of Food Composition (4th revised edition)^10^ and the Follow-up of the Standard Tables of Food Composition (4th revised edition)^11^ for the nutrient content of foods which were not listed in the Chinese Standard Table.

Alcohol consumption was measured with reference to a specific Chinese cup for alcohol. All interviewees were asked to estimate the intake of alcohol and then energy intake from this source was calculated, using the Food Composition Table of China^9^ and added to the total energy intake.

The parameters of interest were total energy, protein, fat (animal, vegetable and marine), carbohydrates, crude fiber, retinol, carotene, vitamin C, vitamin E, sodium, potassium, magnesium, calcium, iron, zinc, copper, phosphorus, saturated fatty acids (SFA), mono-unsaturated fatty acids (MUFA), poly-unsaturated fatty acids (PUFA), oleic acid, linoleic acid, arachidonic acid, linolenic acid, eicosapentaenoic acid (EPA), docosahexaenoic acid (DHA), n-3 PUFAs, n-6 PUFAs, and cholesterol.

### Data analyses and selection of foods

The selection of food items for developing SQFFQs was performed using the same procedure as adopted by Tokudome and his colleagues.^8^ In brief, modified contribution analysis was applied to nutrients of interest.^12^^,^^13^ Each food item was listed according to the intake amount of nutrient. We then selected food items with up to a 90 cumulative percentage contribution. Furthermore, we performed forward multiple regression analysis, and selected food items with up to a 0.90 cumulative square of the multiple correlation coefficient (cumulative R^2^).^14^^-^^16^ Finally, we determined food items for SQFFQ which were selected by either contribution analysis and multiple regression analysis. Some food items with up to 0.90 R^2^ but very small % contribution were excluded, because they may be marginal to total nutrient intake. The foods contributing to less than three nutrients, with relatively small % contributions, were also excluded.

The statistical packages SAS^®^ 6.12 and SPSS^®^ for Windows 10.0 were employed for the calculations (SAS Institute, Cary, NC; SPSS Inc., Chicago, IL). Differences in the means of nutrient intake by area and year were tested by the two-tailed Student’s *t* test.

### Intake frequency and portion size for SQFFQs

The intake frequencies were classified into eight categories: almost never; 1-3 times per month; 1-2 times per week; 3-4 times per week; 5-6 times per week; 1 time per day; 2-3 times per day; and 4 or more times per day. Mean amount of each food intake per person and meal was also calculated, using the 3-day weighed record. Then, the standard portion sizes of each food item per meal were determined, using these mean amounts. Portion sizes consumed were listed in the SQFFQ, with six categories, none, 0.5, 1.0, 1.5, 2.0 and more of the standard portion size. We took pictures of the representative foods with standard portion sizes, and made a food model booklet for the standardization of intake amount.

### Nutrient intake in the urban area

The mean intake of total energy, protein, fat and carbohydrates in Nanjing was compared between 7-day and 3-day records for 1995, and between records for 1995 and 2000 to evaluate variation in nutrient intake.

## RESULTS

### Intake of energy and selected nutrients by area

Mean age of rural subjects was slightly older than urban subjects in males and females (Table 1). Urban subjects were taller, but mean BMI in males did not differ between areas. BMI was slightly larger in the rural females than the urban females.

**Table 1.** Intake of energy and selected nutrients by area and gender.

	Males			Females		
	
	Urban	Rural	p-value	Urban	Rural	p-value
	n=94	n=102		n=104	n=112	
Age	44.0 ± 5.9	45.8 ± 6.4	0.044	40.7 ± 2.9	44.1 ± 6.7	<0.001
Height (m)	1.71 ± 0.05	1.68 ± 0.05	<0.001	1.59 ± 0.05	1.56 ± 0.05	<0.001
Weight (kg)	66.3 ± 8.	64.5 ± 8.1	0.118	56.3 ± 7.5	57.8 ± 9.1	0.187
BMI	22.8 ± 2.7	22.9 ± 2.7	0.743	22.1 ± 2.5	23.8 ± 3.4	<0.001
Energy (kcal)	2324 ± 394	2632 ± 630	<0.001	1877 ± 467	2148 ± 651	<0.001
Protein (g)	92.6 ± 19.6	71.2 ± 17.4	<0.001	64.2 ± 20.2	56.1 ± 20.0	0.004
Fat (g)	56.0 ± 18.0	83.3 ± 45.6	<0.001	69.2 ± 24.2	61.9 ± 36.8	0.091
Animal	19.4 ± 8.8	57.6 ± 32.8	<0.001	25.3 ± 12.4	45.9 ± 31.5	<0.001
Plant	36.6 ± 15.2	25.7 ± 24.4	<0.001	43.8 ± 16.9	15.9 ± 17.9	<0.001
Marine	2.3 ± 1.9	1.0 ± 2.7	<0.001	1.2 ± 1.1	0.4 ± 1.0	<0.001
Carbohydrates (g)	351.9 ± 72.0	373.5 ± 118.4	<0.001	249.4 ± 62.3	339.6 ± 98.5	<0.001
Crude fiber (g)	9.5 ± 3.9	11.6 ± 4.5	<0.001	9.6 ± 4.1	9.0 ± 4.6	0.317
Retinol (ug)	327 ± 493	224 ± 343	0.134	243 ± 321	133 ± 346	0.017
Carotene (mg)	2354 ± 1261	3212 ± 2191	0.011	3090 ± 1675	3366 ± 2926	0.396
Vitamin C (mg)	87 ± 40	95 ± 65	0.339	93 ± 46	83 ± 65	0.194
Vitamin E (mg)	35 ± 15	23 ± 14	<0.001	22 ± 10	17 ± 12	<0.001
Sodium (mg)	1910 ± 607	7318 ± 5275	<0.001	2974 ± 1144	5878 ± 4074	<0.001
Potassium (mg)	1943 ± 46	1673 ± 538	0.001	1530 ± 522	1340 ± 547	0.010
Magnesium (mg)	313 ± 71	329 ± 90	0.213	269 ± 93	262 ± 95	0.622
Calcium (mg)	422 ± 151	414 ± 190	0.752	390 ± 151	311 ± 158	<0.001
Iron (mg)	23.3 ± 8.5	21.3 ± 7.3	0.125	19.4 ± 7.6	19.8 ± 8.1	0.737
Zinc (mg)	12.7 ± 2.6	11.1 ± 2.8	<0.001	10.1 ± 3.5	9.4 ± 3.5	0.175
Copper (mg)	2.2 ± 0.7	2.1 ± 0.8	0.589	2.0 ± 0.7	1.7 ± 0.7	0.001
Phosphorus (mg)	1170 ± 252	1027 ± 246	<0.001	884 ± 265	818 ± 273	0.074
Selenium (*μ*g)	66 ± 21	40 ± 18	<0.001	42 ± 16	26 ± 12	<0.001
SFA (g)	14.3 ± 4.3	31.9 ± 21.4	<0.00	18.9 ± 6.8	24.8 ± 18.2	0.002
MUFA (g)	21.2 ± 6.	37.3 ± 23.5	<0.001	22.7 ± 9.7	26.4 ± 22.1	0.114
PUFA (g)	16.4 ± 7.1	15.4 ± 8.6	0.391	22.9 ± 8.4	10.9 ± 7.1	<0.001
Oleic acid (g)	18.7 ± 5.6	31.2 ± 21.1	<0.001	22.8 ± 8.7	24.6 ± 17.7	0.359
Linoleic acid (g)	13.6 ± 6.2	13.9 ± 7.6	0.829	19.8 ± 7.4	9.7 ± 6.0	0.001
Arachidonic acid (g)	0.06 ± 0.06	0.03 ± 0.03	<0.001	0.06 ± 0.05	0.02 ± 0.03	<0.001
Linolenic acid (g)	2.7 ± 1.0	1.5 ± 1.6	<0.001	3.0 ± 1.2	1.2 ± 1.5	<0.001
EPA (g)	0.03 ± 0.04	0.01 ± 0.01	<0.001	0.03 ± 0.05	0.01 ± 0.02	<0.001
DHA (g)	0.07 ± 0.11	0.01 ± 0.01	<0.001	0.04 ± 0.05	0.01 ± 0.01	<0.001
n-3 PUFA (g)	2.8 ± 1.1	1.5 ± 1.6	<0.001	3.0 ± 1.2	1.2 ± 1.5	<0.001
n-6 PUFA (g)	13.6 ± 6.2	13.9 ± 7.6	0.857	19.9 ± 7.4	9.7 ± 6.0	<0.001
Cholesterol (mg)	62 ± 29	197 ± 145	<0.001	43 ± 25	177 ± 177	<0.001

BMI: body mass index, SFA: saturated fatty acid, MUFA: mono-unsaturated fatty acid, PUFA, poly-unsaturated fatty acid, EPA: eicosapentaenoic acid, DHA: docosahexaenoic acid.

Energy and carbohydrate intake was greater in the rural area, but mean amounts of protein consumed were higher in the urban area. Total fat was consumed more by rural than by urban males. Animal fat (lard) was the major contributor to fat intake in the rural area, whereas plant oil was the major source in the urban area. Marine oil was also taken more in the urban population, although the contribution to total fat intake was minor. Higher intake of saturated fatty acid, MUFA and cholesterol in the rural population was concordantly observed with animal fat intake. On the contrary, more arachidonic acid, linolenic acid, EPA, DHA and n-3 PUFA were consumed in the urban population. Crude fiber consumption was also greater in the rural males, but geographical variation was not clear in females. More carotene and sodium were taken by the rural males, and more sodium but less calcium by the rural females. More potassium was consumed in the urban population. Although selenium was not included in the nutrients of interest for the SQFFQs, we compare its intake between areas, because selenium is an important mineral for cancer etiology.^17^ The mean intake of selenium in the urban area was larger than that in the rural area in both genders.

### Selection of Food items

The total numbers of food items that were consumed by the inhabitants during three days were 415 in the urban, and 211 in the rural area. The food items according to intake contribution of 29 nutrients with up to 90% cumulative contribution and 0.9 cumulative R^2^ are listed in Table 2. The number of urban food items for the cumulative contribution and cumulative R^2^ was greater than for rural items with all nutrients, except for cholesterol intake in the cumulative R^2^. The total numbers of food items with up to 90% cumulative contribution for 29 nutrients were 191 in the urban and 82 in the rural case, and for up to 0.9 cumulative R^2^ were 133 and 56, respectively. Finally, 117 and 76 food items were selected for SQFFQs in the urban and rural areas respectively. Tea was intentionally added, because it is an important dietary factor involved in cancer risk.

**Table 2.** Number of foods contributing to 29 nutrients with up to 90 cumulative % contribution and 0.9 cumulative R^2^ by area.

	Cumulative % contribution			Cumulative R^2^		
	
	Urban	Rural	Common	Urban	Rural	Common
Energy	70	21	14	28	12	5
Protein	86	36	23	26	22	5
Fat	46	12	8	17	3	2
Carbohydrates	34	11	7	8	7	2
Crude fiber	75	36	19	28	19	3
Retinol 1	9	5	4	3	1	1
Carotene	17	14	7	6	3	3
Vitamin C	26	16	8	13	4	4
Vitamin E	53	26	15	11	9	4
Sodium	32	5	5	4	2	2
Potassium	98	43	27	35	22	3
Magnesium	90	37	22	32	17	4
Calcium	87	46	14	26	12	6
Iron	102	43	25	21	19	5
Zinc	89	34	22	26	19	4
Copper	90	36	21	26	17	6
Phosphorus	88	33	24	30	19	5
SFA	38	7	6	3	2	0
MUFA	36	9	8	4	3	1
PUFA	34	13	9	11	7	6
Oleic acid	34	9	7	16	2	1
Linoleic acid	29	12	8	11	7	5
Arachidonic acid	24	6	5	7	5	3
Linolenic acid	17	17	7	3	2	1
EPA	16	5	3	6	3	1
DHA	12	8	4	5	4	1
n-3 PUFA	20	17	10	5	2	2
n-6 PUFA	29	12	9	11	7	5
Cholesterol	28	8	6	3	4	2

SFA: saturated fatty acid, MUFA: mono-unsaturated fatty acid, PUFA, poly-unsaturated fatty acid, EPA: eicosapentaenoic acid, DHA: docosahexaenoic acid.

### List of food items

Rice was the most important contributing food for energy intake in both urban and rural areas (Table 3). Salad oil, flour, soybean oil and pork followed it in the urban area. In contrast, lard, corn and dried noodles contributed more to energy intake in the rural area, in addition to pork. Rice was also contributed most to protein and carbohydrate intake in both areas. Contributing food items for fat intake were quite different between the two areas. In the urban area the major items were vegetable oils, such as salad oil and soybean oil, while those in the rural area were of animal origin, such as lard and pork. Rapeseed oil was taken more in the rural area.

**Table 3.** Percentage contribution of the top 10 foods for energy, protein, fat and carbohydrates in urban and rural areas.

Energy				Protein				Fat				Carbohydrates			
			
Urban		Rural		Urban		Rural		Urban		Rural		Urban		Rural	
Rice	36.9	Rice	39.5	Rice	23.1	Rice	34.4	Salad oil	22.1	Lard	45.8	Rice	57.1	Rice	59.6
Salad oil	6.9	Lard	14.2	Lean pork	7.2	Pork	6.5	Soybean oil	17.1	Pork	16.4	Flour	8.7	D noodles	5.8
Flour	5.9	Pork	5.3	Egg	5.0	Egg	4.3	Pork	9.5	Rape oil	11.7	FD noodles	2.9	Corn	5.7
Soybean oil	3.8	Corn	3.8	Flour	5.0	D noodles	4.2	Egg	3.8	Rice	3.8	Sucrose	2.6	Corn porridge	4.4
Pork	3.6	Dried noodles	3.6	Herring	3.8	Corn	3.5	Lean pork	3.7	Egg	2.3	Noodles	2.4	Flour	3.6
Lean pork	2.0	Corn porridge	3.4	Pork	3.6	Bean curd	3.0	Pork steak	2.8	Fat pork	1.8	Steamed bread	2.0	Hybrid rice	3.0
Fine dried noodles	2.0	Rape oil	3.3	Chicken	2.5	Flour	2.9	Rice	2.6	Bean curd*	1.7	Apple	1.9	Corn starch	2.9
Egg	1.8	Flour	2.5	Bean curd	2.2	Greengrocery	2.7	Peanut	2.3	Corn	1.6	Vermicelli	1.2	Noodles**	2.4
Noodles	1.5	Corn starch	2.1	Pork steak	1.5	Corn porridge	2.6	Sausage	1.8	Corn porridge	1.6	Glutinous rice***	1.1	Ground rice	1.1
Steamed bread	1.4	Wine	2.0	Greengrocery	1.5	Bean card*	2.3	Rape oil	1.5	Bean curd	1.6	Dough stick****	0.8	Noodles	0.9

D: dried, FD: fine dried.

*Thin sheet; **enriched; ***polished; ****fried twis

Rice and greengroceries similarly contributed to crude fiber intake in both areas, as well as corn in the rural population (data not shown in tables). A greater variety of vegetables contributed to the top ten foods for crude fiber intake in the urban area.

### Nutrient coverage in the SQFFQs

The selected food items in the SQFFQ for the urban area covered 18 nutrients with up to 90% of the total intake, except crude fiber, vitamin A, magnesium, iron, copper, PUFA, n6-PUFA, linoleic acid, arachidonic acid, EPA and DHA (Table 4). The SQFFQ for the rural area covered most of nutrients with up to 90% of the total intake, except for EPA and DHA. The lowest coverage percentage was for DHA in the rural SQFFQ, at 77%.

**Table 4.** Nutrient coverage (%) of the foods in the SQFFQs.

	Urban	Rural
Energy	94	97
Protein	92	93
Fat	90	98
Carbohydrates	96	97
Crude fiber	88	91
Carotene	97	93
Vitamin A	84	97
Vitamin C	94	95
Vitamin E	91	93
Potassium	91	92
Sodium	94	99
Calcium	92	92
Magnesium	88	93
Iron	88	94
Zinc	92	94
Copper	89	92
Phosphorus	91	94
Cholesterol	91	97
SFA	91	99
MUFA	91	99
PUFA	87	97
*n*3 PUFA	92	97
n6 PUFA	86	97
Oleic acid	92	99
Linoleic acid	86	97
Arachidonic acid	88	93
Linolenic acid	93	97
EPA	81	82
DHA	85	77

SFA: saturated fatty acid, MUFA: mono-unsaturated fatty acid, PUFA, poly-unsaturated fatty acid, EPA: eicosapentaenoic acid, DHA: docosahexaenoic acid.

### Comparison of nutrient intake in the urban area

The mean intake of total energy, protein, fat and carbohydrates in Nanjing did not differ between 7-day and 3-day records in 1995 (Table 5). Furthermore, no significant differences between 3-day records of 1995 and 2000 were found. Although protein intake in 2000 tended to be higher than that in 1995, the proportional increase was relatively small (9.2%).

**Table 5.** Comparison of mean intake and its standard deviation of total energy, protein and fat in Nanjing between 7-day and 3-day records, and between 3-day records for 1995 and 2000.

	7-day record	3-day record		p-value*	p-value**
	
	1995	1995	2000		
	n=198	n=198	n=50		
Energy (kcal)	2094 ± 467	2099 ± 456	2138 ± 189	0.916	0.615
Protein (g)	78.0 ± 24.1	77.3 ± 23.2	84.4 ± 24.8	0.793	0.056
Fat (g)	63.0 ± 22.1	61.9 ± 25.2	67.0 ± 31.3	0.626	0.278
Carbohydrates (g)	298.7 ± 81.6	305.0 ± 79.5	298.6 ± 78.2	0.440	0.607

*Between 7-day and 3-day records for 1995.

**Between 3-day records for 1995 and 2000.

## DISCUSSION

In the present study we observed that energy intake by inhabitants in a rural area is greater than by urban inhabitants in the same province of China. Higher consumption of carbohydrates and fat, especially lard, in both genders, and alcohol in males, of the rural area was a major contributor to this difference. A nationwide survey of 1992 showed the national average of energy intake to be higher in urban than in rural areas, especially in the population with middle and high income,^7^ but in the present study the opposite was the case. However, the present findings obtained in Jiangsu Province are concordant with the results of the Nationwide Survey for the same province, although the data of the nationwide survey was obtained 3 and 8 years before the present urban and rural survey, respectively. The total energy intake in males was only 4.1% lower (2,423 kcal) in the present urban area and 3.3% higher (2,549 kcal) in the rural area than those in the representative urban and rural areas of the same province by nationwide survey. The mean intakes of major nutrients in the present study were 16.6% higher in the urban area and 3.3% lower in the rural area for protein; 11.1% higher and 6.7% lower for carbohydrate; 1.1% higher and 31.4% lower for crude fiber; but 38.7% lower and 20.9% higher for fat, compared with the respective figures from the nationwide survey. The small differences in intake of total energy, protein and carbohydrate between the present study and the nationwide survey suggest the present results are appropriate to estimate nutrient intake in the study areas. Fat intake revealed an opposite trend between the surveys, and further validity testing, such as examination of serum lipid levels, should be conducted to evaluate geographical variation, because there are no available reports other than that of the nationwide survey.

We observed significant differences of nutrient intake between the urban and rural areas for protein, carbohydrates, sodium and selenium in both males and females. These differences were concordantly revealed in the urban and rural areas of Jiangsu Province on the nationwide survey, except fat intake.^7^ However, sodium intake in the urban males was 73.9% lower in the urban area and 18.5% lower than those by nationwide survey. As we did not examine the urine sodium level to validate the estimation of its intake and no report is available for its validation, further tests are also needed to evaluate the accuracy of the present results. Most other minerals revealed relatively small differences (within 20%) in their intake between the present study and the nationwide survey, except for selenium intake, this being 35.8% higher in the urban area of the present study.

We observed the present urban inhabitants consumed more plant oil, but less animal oil than the rural inhabitants. This dietary habit may be influenced by a higher health consciousness in the urban population. Females consumed more plant oil than males in this urban area, being partially due to higher consumption of nuts and cookies, in which plant oils are important constituents. Adoption of the same method to collect dietary records and estimate nutrient intake for both genders and study areas, however, should mean a low potential for systemic error in this difference.

Animal fat (lard) intake in the present rural area was much higher than that of national average level in the rural area of Nationwide Survey, including the case of Jiangsu Province itself. It is of interest that the serum lipid level among healthy inhabitants of Shanghai, metropolitan city, is known to be slightly higher in urban than in rural inhabitants.^18^ These facts suggest a potential that the high intake of lard in the present rural area is an area-specific dietary habit. We also observed higher fat intake in the rural area of Jinghu county, Jiangsu Province, in 1990, compared with that in Nanjing (personal communication). Rural populations generally work hard and require more energy intake with more grain than urban populations. Rural people in the study area take more lard with grain instead of vegetables and animal protein, due to their poor economic condition. Females proportionally consume more plant oil than males in the urban areas, and males more animal oil than females in the rural area.

We here used contribution analysis and multiple regression analysis to select representative food items for stable food intake. In the former case this is based on absolute food and nutrient intake, which indicates the contributed percentage of the food to nutrients.^12^^,^^13^ Hence, this procedure is especially suitable for studies to classify associations with absolute nutrient intake. Selection by multiple regression analysis, in contrast, is based on the variance of nutrient intake.^14^^-^^16^ Although the number of selected food items by cumulative R^2^ was smaller than that by cumulative percentage contribution, multiple regression analysis is efficient for categorization. Therefore the combination of these two methods for food selection provides us with more suitable SQFFQs for diet assessment and provision of accurate dietary information.

We selected 117 and 76 food items in the urban and rural areas, respectively. Most of these were consumed by inhabitants of both areas, but some were area-specific, such as yoghurt in the urban area; and lard, rape oil, bacon and hybrid rice in the rural area. Such differences in food items consumed emphasize the necessity to make area-specific SQFFQs to estimate cancer risk from dietary factors in the study area. Though it is difficult to say that the selected foods represent all intakes of nutrients, they cover about 90% of the total nutrient intake. Therefore, the information provided by SQFFQs with their use would be expected to be representative and in line with conventional dietary data in the study population. As the next step, validity and reproducibility tests are now needed to evaluate SQFFQ appropriateness and characteristics.^4^^,^^19^

In the present study data for the urban area collected in 1995 were compared with rural data for 2000. However, we used a standardized procedure for data collection in both areas, and most of staffs were involved in both surveys and a comparison of the intake of energy and major-nutrients between the subjects in 1995 and newly-recruited subjects in 2000, demonstrated no significant differences. Protein intake showed a tendency to rise during the 5 years, but its proportional increase was relatively small. We also compared the results of 7-day and 3-day weighed record methods and found no selection bias for mean intake of energy and macronutrients.

In summary, in the present investigation we clarified common and specific intake of foods and 29 nutrients in urban and rural areas of Jiangsu Province, China, for adoption in area-specific SQFFQs. We are now planning to determine whether a combined SQFFQ that includes the urban and rural food items would allow better assessment of cancer risk and benefit of dietary factors, by validity and reproducibility testing.
